# Erosion of estimated genomic breeding values with generations is due to long distance associations between markers and QTL

**DOI:** 10.1186/s12711-025-00963-5

**Published:** 2025-03-21

**Authors:** Didier Boichard, Sébastien Fritz, Pascal Croiseau, Vincent Ducrocq, Thierry Tribout, Beatriz C. D. Cuyabano

**Affiliations:** 1 Université Paris-Saclay, INRAE, AgroParisTech, GABI, 78350 Jouy-en-Josas, France; 2 Eliance, 75012 Paris, France

## Abstract

**Background:**

Most validation studies of genomic evaluations on candidates (prior to observing phenotypes) present inflation of their predicted breeding values, i.e., regression coefficients of their later observed phenotypes on the early predictions are smaller than one. The aim of this study was to show that this inflation pattern reflects at least partly long-distance associations between markers and quantitative trait loci (QTL) in the reference population and to propose methods to estimate the corresponding “erosion” coefficient.

**Results:**

Across-chromosome linkage disequilibrium (LD) is observed in different dairy cattle breeds, being a result from limited effective population size and from relationships within the reference population. Due to this long distance LD, the estimated SNP effects capture non-zero contributions from distant QTLs, some located on other chromosomes than the SNP itself. Therefore, corresponding SNP effects are partly lost in the next generations and we refer to this loss as “erosion”. With the concept of QTL contribution to SNP effects derived from mixed model equations, we show with simulation that this long range LD explains 6–25% of the variance of the estimated genomic breeding values, a proportion that is unchanged when the evaluation model includes a residual polygenic effect. Two methods are proposed to predict this erosion factor assuming known simulated QTL effects. In Method 1, one generation of progeny is simulated from the reference population and the GEBV of these progeny based on SNP effects estimated in this newly simulated generation are regressed on the GEBV of the same progeny based on SNP effects estimated in the reference population. In Method 2 all the QTL contributions to SNP effects are regressed based on SNP-QTL recombination rates and summed to predict the GEBV at the next generation. The regression coefficient of the GEBV based on eroded contributions on the raw GEBV is also an estimate of erosion. An illustration is given with the French Normande female reference bovine population in 2021, showing erosion factors ranging from 0.84 to 0.87.

**Conclusion:**

Accounting for erosion is important to avoid inflation and biased predictions. The ways to both reduce inflation and to correct for it in the prediction are discussed.

## Background

In genomic evaluation [1], SNP effects are estimated in a reference sample and applied to candidates for selection. This method is extensively used to identify the best candidates at an early stage of their life or yet without phenotypic information. The initial and still frequent reasoning of this method is that SNPs are in close linkage disequilibrium (LD) with causal mutations (or quantitative trait nucleotides, QTN) and therefore, are good proxies for these mutations. Implicitly, this assumes that estimated SNP effects reflect effects of the neighbouring causal mutations only. Under this assumption, associations observed in the reference should be conserved in the next generation as short distance linkage disequilibrium is only slowly eroded by recombinations.

However, it is well known that genomic evaluation efficiency is highly dependent on the close relationship of the candidates to the reference sample because an important part of information is the relationship between individuals captured by markers [2–5]. This is especially true when the same prior is assumed for all SNP effects, as in genomic BLUP (GBLUP, [6]) (or, equivalently, in Single Nucleotide Polymorphism BLUP (SNP-BLUP, [2, 7])), which is the standard method used in routine evaluation. Many studies have shown the limited gain in accuracy in multi-breed evaluation [8, 9], illustrating that distant reference populations are either not or little informative. Other studies have shown a decrease in accuracy over generations when the reference sample is not updated [10, 11] and that the absence of parents in the reference sample directly influences the prediction accuracy of the selection candidates [12]. Validation studies of genomic evaluations on young candidates (prior to observing phenotypes) are generally based on the regression of the later observed performances on the early predictions. These studies frequently observe an inflation pattern, i.e., a regression coefficient that is systematically lower than 1 (e.g., [13–16]). Inflation means that later performances of the candidates with the highest predicted genomic estimated breeding values are below those initially predicted and later performances of the worst candidates, if any (given that these candidates are usually not included in breeding programs), are above those initially predicted. All these results suggest than SNP effect estimates erode (or do not persist) as the distance between candidates and reference sample increases.

One plausible interpretation is the existence of long-range LD, even across different chromosomes. Consequently, many markers may capture partial effects of supposedly unlinked QTL. Although average long-distance LD is low and much lower than short-distance LD, the number of long-distant variants is considerably higher and their combined effects may account for some proportion of the genomic prediction.

The objective of this study was (a) to demonstrate that with GBLUP, markers capture part of the effects of distant QTL due to long-distance LD, and (b) to propose two methods based on simulation to estimate the erosion factor specific to a reference population. Based on its conclusions, this article also suggests how erosion factors can be used in practice to adjust genomic predictions (GEBV) computed by GBLUP, and how the impact of erosion can be limited.

## Methods

In this section, we first describe a method to compute the contribution of each QTL (assumed to be known) to each SNP effect. These contributions can be used to generate partial GEBV defined by the distance between each QTL to each SNP, and these partial GEBV can be used to measure the impact of long distance LD. This approach is presented first for a model without polygenic effect, and then extended to a model with a polygenic effect.

Then, we present two methods based on QTL simulation in real populations to estimate the erosion factors. The extent of LD across chromosomes is presented in six different French cattle breeds of very different size. To one of them, the Normande breed of medium effective size (Ne = 93, [17]), the proposed methods in this article are applied, and the corresponding QTL simulation design is presented.

### Decomposition of genomic breeding values into QTL contributions to SNP effects

The strategy used to compute erosion relies on the determination of the contribution of each QTL to each SNP effect, as follows, assuming that the QTL are known. Ignoring the fixed effects and a potential polygenic effect, the SNP-BLUP model is:

$$\mathbf{y}=\mathbf{M}\mathbf{s}+\mathbf{e},$$

where $\mathbf{y}$ is the vector of N phenotypes, $\mathbf{M}$ the (N x ns) matrix of centered and scaled genotypes, $\mathbf{s}$ the vector of ns SNP effects assumed to be normally distributed $N(\mathbf{0},\mathbf{I} {\upsigma }_{\text{s}}^{2})$, and $\mathbf{e}$ the vector of residuals assumed to be normally distributed $N(\mathbf{0},\mathbf{I} {\upsigma }_{\text{e}}^{2})$.

The corresponding SNP-BLUP equations can be written as:

1 $$\left[\mathbf{M}\mathbf{^{\prime}}\mathbf{M}+\uplambda \mathbf{I}\right]\widehat{\mathbf{s}}={\mathbf{M}}^{\mathbf{^{\prime}}}\mathbf{y},$$

with $\mathbf{I}$ the identity matrix and $\uplambda = \sigma_{e}^{2}/ {\sigma }_{s}^{2}$.

We assume that the phenotype is the result of the sum of nq QTL effects and an error term, such that $\mathbf{y}=\mathbf{P}\mathbf{q}+{\varvec{\upvarepsilon}}$ with $\mathbf{P}$ the (N x nq) matrix of QTL genotypes and $\mathbf{q}$ the vector of true QTL effects. Therefore, Eq. (1) can be rewritten as:

$$\widehat{\mathbf{s}}={\left[\mathbf{M}{^{\prime}}\mathbf{M}+ {\varvec{\uplambda}}\mathbf{I}\right]}^{-1} {\mathbf{M}}^{{^{\prime}}}\left(\mathbf{P}\mathbf{q}+{\varvec{\upvarepsilon}}\right).$$

Let us denote $\mathbf{C}={\left[\mathbf{M}\mathbf{^{\prime}}\mathbf{M}+\uplambda \mathbf{I}\right]}^{-1}$ the inverse of coefficient matrix. The contribution of QTL j to SNP effect i is:

2 $${\text{f}}_{\text{ij}}= {\mathbf{c}}_{\mathbf{i}}{\mathbf{M}}^{\mathbf{^{\prime}}}{\mathbf{p}}_{\mathbf{j}}{\text{q}}_{\text{j}},$$

where ${\mathbf{c}}_{\mathbf{i}}$ is row i (corresponding to SNP i) of $\mathbf{C}$ and ${\mathbf{p}}_{\mathbf{j}}$ the vector of genotypes for QTL j.

Only expected contributions were considered assuming QTL effects are known and ignoring the residual component. Therefore, the situation is ideal to estimate SNP effects. The only impact of the heritability is the prior variance ratio in the equations, which leads to regress the SNP effects and therefore, the QTL contributions to SNPs.

The estimated genomic value is the sum of the nq x ns such contributions multiplied by the individual SNP genotypes. These contributions are difficult to analyse individually because they have strong covariances and the sum of their individual variances is much smaller than the genomic variance. Alternatively, they can be summed by classes to define partial genomic values according to the SNP-QTL distance (d). In this study, five classes were defined: (1) d = 0 (the SNP is the QTL); (2) d < 5 Mb; (3) 5 < d < 20 Mb; (4) the QTL and the SNP are on the same chromosome but d > 20 Mb; (5) the QTL and the SNP are on different chromosomes.

To study the distribution of these contributions, QTL effects have to be known. Therefore, they were simulated according to different scenarios. The partial genomic values were then used to estimate their relative contribution to the whole genomic value and the covariances between them.

### Decomposition of genomic breeding values in the case of a residual polygenic effect

Many evaluation models include both genomic and residual polygenic effects. The same strategy can be used, but with more computations. Again, ignoring fixed effects, the SNP-BLUP model can be written as:

$$\mathbf{y}=\mathbf{Z}\mathbf{u}+\mathbf{M}\mathbf{s}+ \mathbf{e},$$

where $\mathbf{u}$ is the vector of residual polygenic effect which are normally distributed $N(\mathbf{0},\mathbf{A} {\upsigma }_{\text{u}}^{2})$.

The corresponding system of equations is the following:

3 $$\left[\begin{array}{cc}{ \mathbf{Z}^{\prime}}\mathbf{Z}+\upkappa {\mathbf{A}}^{-1}& {\mathbf{Z}^{\prime}}\mathbf{M}\\ {\mathbf{M}^{\prime}}\mathbf{Z}&{\mathbf{M}^{\prime}}\mathbf{M}+\uplambda \mathbf{I} \end{array}\right]\left[\begin{array}{c}\widehat{\mathbf{u}}\\ \widehat{\mathbf{s}}\end{array}\right]= \left[\begin{array}{c}{\mathbf{Z}^{\prime}}\mathbf{y}\\ {\mathbf{M}^{\prime}}\mathbf{y}\end{array}\right],$$

with $\mathbf{Z}$ being the incidence matrix linking the records $\mathbf{y}$ to $\mathbf{u}$, and $\upkappa = {\sigma}_{e}^{2}/\sigma_{u}^{2}$ the corresponding variance ratio. The $\mathbf{u}$ equations can be absorbed into the $\mathbf{s}$ equations, by replacing $\widehat{\mathbf{u}}$ by $\widehat{\mathbf{u}}=\boldsymbol{ }{\left[{\mathbf{Z}}^{\mathbf{^{\prime}}}\mathbf{Z}+\upkappa {\mathbf{A}}^{-1}\right]}^{-1}$(${\mathbf{Z}}^{\mathbf{^{\prime}}}\mathbf{y}-{\mathbf{Z}}^{\mathbf{^{\prime}}}\mathbf{M}\boldsymbol{ }\widehat{\mathbf{s}}$). This results in the following formula after replacing $\mathbf{y}$ by $\mathbf{P}\mathbf{q}+{\varvec{\upvarepsilon}}$:

$$\left[{\mathbf{M}}^{{^{\prime}}}\left(\mathbf{I} -\mathbf{Z}{\left({\mathbf{Z}}^{{^{\prime}}}\mathbf{Z}+\upkappa {\mathbf{A}}^{-1}\right)}^{-1}{\mathbf{Z}}^{{^{\prime}}}\right)\mathbf{M}+\uplambda \mathbf{I}\right] \widehat{\mathbf{s}}={\mathbf{M}}^{{^{\prime}}}\left(\mathbf{I} -\mathbf{Z}{\left({\mathbf{Z}}^{{^{\prime}}}\mathbf{Z}+\upkappa {\mathbf{A}}^{-1}\right)}^{-1}\mathbf{Z}{^{\prime}}\right) (\mathbf{P}\mathbf{q}+{\varvec{\upvarepsilon}})$$

Let us denote ${\mathbf{C}}^{*}$ the inverse of the coefficient matrix after absorption

$${{\mathbf{C}}^{*}=\left[{ \mathbf{M}}^{{^{\prime}}}\left(\mathbf{I} -\mathbf{Z}{\left({\mathbf{Z}}^{{^{\prime}}}\mathbf{Z}+\upkappa {\mathbf{A}}^{-1}\right)}^{-1}{\mathbf{Z}}^{{^{\prime}}}\right)\mathbf{M}+\uplambda \mathbf{I} \right]}^{-1}$$

and ${\mathbf{M}}^{*}$ the adjusted genotype matrix after absorption

$${{\mathbf{M}}^{*}}^{{^{\prime}}}={\mathbf{M}}^{{\prime}}\left(\mathbf{I} -\mathbf{Z}{\left({\mathbf{Z}}^{{^{\prime}}}\mathbf{Z}+\upkappa {\mathbf{A}}^{-1}\right)}^{-1}{\mathbf{Z}}^{{^{\prime}}}\right).$$

Then, as in (2), the contribution of QTL j to each SNP effect i can be written as:

4 $${\text{f}}_{\text{ij}}= {\mathbf{c}}_{\mathbf{i}}^{*}{\mathbf{M}}^{*{^{\prime}}}{\mathbf{p}}_{\mathbf{j}}{\text{q}}_{\text{j}},$$

where ${\mathbf{c}}_{\mathbf{i}}^{\mathbf{*}}$ is row i of ${\mathbf{C}}^{*}$. Therefore, the same contribution approach can be used with a residual polygenic effect.

### Methods to estimate erosion between generations

In this paragraph, we present two methods based on QTL simulation in real populations, in order to estimate the consequence of erosion on GEBV of the next generation. The erosion of the genomic breeding values has two distinct components: (a) a component that is characteristic of the reference population itself, and (b) a component that is specific to each candidate and its genetic distance from the reference population (which determines the conservation of SNP x QTL covariances, among the candidates). In this paragraph, we assume that the parents of the candidates belong to the reference population and we focus on the erosion between the reference population and the candidates in the next generation.

The extent of long-distance LD in the reference population is influenced by the effective population size (Ne). Notably, when Ne is small, a non-zero LD baseline persists. More importantly, the level of long-distance LD is also dependent on the genetic relationship within the reference population, which can be larger than the relationship in the overall population. A higher average relationship between individuals within the reference population results in more long-distance LD.

The theoretical derivation of the erosion factor $\varrho$ is not presented here and requires additional investigation. Some proposals were made by [18] and [19] to account for erosion in the computation of reliability, but not to directly account for it in genomic estimated breeding values. In this article, we present two simulation-based approaches which offer practical, intuitive, and effective means to address the erosion phenomenon and to estimate $\varrho$.

### Method 1: by simulating a new generation

The reference population R of the breed for a given trait is considered with its SNP genotypes ${\mathbf{M}}_{\mathbf{r}}$. Nq QTL are simulated in this reference population by sampling SNP which are thereafter excluded from ${\mathbf{M}}_{\mathbf{r}}$ and therefore from the analysis. Expectations of SNP effects are computed by $\widehat{{\mathbf{s}}_{\mathbf{r}}}={({\mathbf{M}}_{\mathbf{r}}^{\prime}{\mathbf{M}}_{\mathbf{r}}\text{+} \lambda \mathbf{I})}^{-1}{\mathbf{M}}_{\mathbf{r}}^{\prime}{\mathbf{P}}_{\mathbf{r}}\mathbf{q}$, assuming the same previous notations with subscript r standing for the reference population.

A new generation, N, is then simulated. In a first step, parents are sampled and mated in the reference population, either at random or following a predefined design. In the second step, gametes are produced by sampling parental chromosomes with 0.5 probability and recombination events according to the genetic map, assuming 1 Mb corresponds to 1 cM. The expected genomic values $\widehat{{\mathbf{g}}_{\mathbf{n}\mathbf{r}}}$ of this new generation are obtained from its genotypes ${\mathbf{M}}_{\mathbf{n}}$ and from the SNP effects estimated in the reference population: $\widehat{{\mathbf{g}}_{\mathbf{n}\mathbf{r}}}= {\mathbf{M}}_{\mathbf{n}}\widehat{{\mathbf{s}}_{\mathbf{r}}}$. Assuming phenotypes are then known in this new generation, new SNP effects can be estimated from population N only as $\widehat{{\mathbf{s}}_{\mathbf{n}}}={({\mathbf{M}}_{\mathbf{n}}^{\prime} \, {\mathbf{M}}_{\mathbf{n}}\text{ + } \lambda \mathbf{I})}^{-1}{\mathbf{M}}_{\mathbf{n}}^{\prime}{\mathbf{P}}_{\mathbf{n}}\mathbf{q}$, and a new set of expected genomic values is obtained from these new SNP estimates $\widehat{{\mathbf{g}}_{\mathbf{n}}}= {\mathbf{M}}_{\mathbf{n}}\widehat{{\mathbf{s}}_{\mathbf{n}}}$. These new SNP effects are different from the previous ones if the covariances (i.e., the LD) between SNP and QTL $\mathbf{M}_\mathbf{r}^{\prime} \mathbf{P}_\mathbf{r}$ and $\mathbf{M}_\mathbf{n}^\prime {\mathbf{P}}_{\mathbf{n}}$ differ. A large change in the covariances between SNP ${\mathbf{M}}_{\mathbf{r}}^{\prime}{\mathbf{M}}_{{\varvec{r}}}$ and ${\mathbf{M}}_{\mathbf{n}}{^\prime}{\mathbf{M}}_{\mathbf{n}}$ (i.e., a large change in LD between SNP) may also affect the results but probably to a lesser extent.

From these two sets of estimated genomic values, an estimate of the erosion $\varrho$ between the two generations is obtained through a regression analysis:

5 $$\widehat{{\mathbf{g}}_{\mathbf{n}}}= \mathbf{1}{\upmu }_{1}+{ \varrho }\widehat{{\mathbf{g}}_{\mathbf{n}\mathbf{r}}}+{\mathbf{e}}_{\mathbf{1}}.$$

The procedure can be summarized as follows:

- Define a reference population, sample nq QTLs among the SNPs
- Estimate SNP effects ($\widehat{{\mathbf{s}}_{\mathbf{r}}}$) in the reference
- Sample the parents in the reference, define matings and number of progeny per mating
- Sample SNP (${\mathbf{M}}_{\mathbf{n}}$) and QTL (${\mathbf{P}}_{\mathbf{n}}$) genotypes of progeny, according to genetic map
- Compute $\widehat{{\mathbf{g}}_{\mathbf{n}\mathbf{r}}}$ of progeny with genotypes ${\mathbf{M}}_{\mathbf{n}}$ and SNP effects $\widehat{{\mathbf{s}}_{\mathbf{r}}}$ from the reference
- Estimate SNP effects ($\widehat{{\mathbf{s}}_{\mathbf{n}}}$) in the generation of progeny
- Compute $\widehat{{\mathbf{g}}_{\mathbf{n}}}$ of progeny based on ${\mathbf{M}}_{\mathbf{n}}$ genotypes and $\widehat{{\mathbf{s}}_{\mathbf{n}}}$ SNP effects
- Regress $$\widehat{{\mathbf{g}}_{\mathbf{n}}}$$ on $$\widehat{{\mathbf{g}}_{\mathbf{n}\mathbf{r}}}$$.

### Method 2: by regressing contributions of QTL to marker effects

As above, the real population R of the breed for a given trait is used to simulate nq QTL and ${\mathbf{M}}_{\mathbf{r}}$ is the matrix of remaining SNPs. All contributions ${\text{f}}_{\text{ij}}$ of the QTL j to the effect of SNP i are computed as shown in Eq. (2). $\widehat{{\mathbf{g}}_{\mathbf{r}}}$ in the reference population is the sum of all contributions or, equivalently, the sum of the SNP genotypes times the SNP effects:

$$\widehat{{\mathbf{g}}_{\mathbf{r}}}={\mathbf{M}}_{\mathbf{r}}\mathbf{f}\mathbf{1}_{\mathbf{q}}= {\mathbf{M}}_{\mathbf{r}}\widehat{{\mathbf{s}}_{\mathbf{r}}},$$

with $\mathbf{1}_{\mathbf{q}}$ being a vector of 1’s of size q.

At the next generation, all ${\text{f}}_{\text{ij}}$ are regressed according to the genetic map, with coefficients $(1-{\text{r}}_{\text{ij}})$ varying from 1 to 0.5, ${\text{r}}_{\text{ij}}$ being the recombination rate between the QTL i and SNP j:

6 $${\text{h}}_{\text{ij}}= {(1-\text{r}}_{\text{ij}}) {\text{ f}}_{\text{ij}}.$$

New eroded genomic values ($\widehat{{\mathbf{g}}_{\mathbf{e}}}$) are the sum of all regressed contributions

$$\widehat{{\mathbf{g}}_{\mathbf{e}}}={\mathbf{M}}_{\mathbf{r}}\mathbf{h}\mathbf{1}_{\mathbf{q}}.$$

Finally another estimate of the erosion factor, ${\varrho }_{2}$, is obtained through the following regression analysis:

7 $$\widehat{{\mathbf{g}}_{\mathbf{e}}}=\mathbf{1}{\mu }_{2}+ {\varrho }_{2}\widehat{{\mathbf{g}}_{\mathbf{r}}}+{\mathbf{e}}_{\mathbf{2}}.$$

Note that $\varrho$ and ${\varrho }_{2}$ are different because $\varrho$ is the erosion for a progeny and ${\varrho }_{2}$ is the erosion for one meiosis. Therefore $\left(1-\varrho \right)=2 \left(1-{\varrho }_{2}\right)$ and an estimate of $\varrho$ based on method 2 is $\varrho =2 {\varrho }_{2}-1$.

The procedure can be summarized as follows:

- Define a reference population, sample nq QTLs among the SNPs
- Compute QTL contributions to SNP effects (${\text{f}}_{\text{ij}}$) in the reference
- Compute $\widehat{{\mathbf{g}}_{\mathbf{r}}}$ in the reference by summing all the contributions
- Compute eroded QTL contributions (${\text{h}}_{\text{ij}}$) to SNP effects, based on recombination rates
- Compute eroded reference genomic values $\widehat{{\mathbf{g}}_{\mathbf{e}}}$ by summing all the eroded contributions
- Regress $$\widehat{{\mathbf{g}}_{\mathbf{e}}}$$ on $$\widehat{{\mathbf{g}}_{\mathbf{n}}}$$.

### Populations used to estimate long distance linkage disequilibrium

In this paragraph, we present six different dairy cattle populations used to measure LD across chromosomes. LD between SNP and QTL was assumed to be the same as between SNP. Because we are interested in long distance LD, we considered only pairs of SNP on different chromosomes. LD was measured in the female reference populations of 6 French dairy cattle breeds (Holstein, Montbeliarde, Normande, Abondance, Tarentaise, Vosgienne) with the data available in 2021. Animals were genotyped with various low or medium-density chips and imputed to 50 k scale with FImpute [20], for a total number of 53,469 SNP distributed over the 29 autosomal chromosomes. Only SNP with a minor allele frequency over 0.01 within breed were considered. The size of these reference populations varied from 2617 to 362,363. It is worth noting that Vosgienne, Tarentaise, and Abondance are regional mountain breeds, whereas Montbeliarde and Normande are large national populations (18 and 7% of the French dairy herd, respectively). On the other hand, the international Holstein breed accounts for 70% of the French dairy cattle population. For our analysis, we selected one out of every 20 SNP of the Illumina EuroGMD BeadChip, resulting in a sample of ~ 3 million r^2^ values for each breed (vs ~ 1.2 billion in total for all SNPs). Rather than the mean ${\text{r}}^{2}$ which is not relevant here, we are more interested in the distribution of the r values (especially those deviating from zero), because the contribution of a given QTL to a given SNP effect is proportional to r.

### Simulation study

In this paragraph, we present the QTL simulation used to show the impact of long distance LD, and to estimate erosion factors with the two proposed methods. Among the six previous populations, we selected the Normande population, i.e., a medium size population to assess the impact of long-distance LD on SNP effects and to estimate the erosion factor. It comprised 69,220 genotyped cows with records. Only the first 5 chromosomes (comprising a total of 13,842 SNP) were considered and nq = 100, 200 or 500 additive QTLs were simulated by sampling at random nq SNPs with MAF > 0.01. QTL effects were independently drawn from a normal distribution $N\left(\mathbf{0}, {\mathbf{I}\upsigma }_{\text{s}}^{2}\right)$ and residuals were drawn from a normal distribution $N\left(\mathbf{0}, {\mathbf{I}\upsigma }_{\text{e}}^{2}\right)$ to generate a heritability of either 0.3, 0.5, or 0.99. All ns x nq contributions were computed according to Eq. (2) and indicators described below were derived from these contributions. In a preliminary analysis with 200 QTL and h^2^ = 0.3, the QTL were kept in the analysis. In all subsequent analyses, causal variants were excluded from the model and, accordingly, class 1 was empty. Because MAF of QTL are likely to be lower than MAF of SNP selected on a chip, an alternative scenario was also tested with 200 QTL with MAF < 0.10.

For each model, the following indicators were computed in this reference population: (a) the portion of variance explained by each class of partial genomic values, expressed in percentage of the sum of these contributions (note that this indicator does not account for the 6 covariances between classes); (b) the correlations between partial genomic values.

In a second step, a residual polygenic effect explaining 20% of the total genetic variance was included in the model. The same indicators were produced.

In a third step, methods 1 and 2 were applied to the simulated dataset for 200 QTL. In method 1, a new generation was created by sampling 1000 sires and 50,000 dams at random in the reference population. Each sire had 50 progeny, while each dam had one progeny, resulting in a new generation of 50,000 animals. For both methods 1 and 2, the recombination rates were based on ARS-UCD1.2 bovine genome assembly, assuming 1 cM per megabase within chromosome, and 50% recombination rate between chromosomes.

For all analyses, thirty replicates were simulated.

## Results

Table 1 presents different indicators of LD values across chromosomes in the reference populations of six French dairy cattle breeds. Average ${\text{r}}^{2}$ values were small, which may suggest at first sight that across chromosomes effects are negligible. These means decreased when the size of the reference population, number of females in the breed, and effective size of the breed increased. It is important to remark that the parameter of interest here is not ${\text{r}}^{2}$ but $\text{r}$, as the impact of a QTL on a SNP effect is proportional to $\text{r}$. The proportion of SNP pairs with $\left|\text{r}\right|$ values greater than 5% varied from 1.9 to 34%, depending on the breed. The larger the reference population, the lower this proportion. Nevertheless, even with a few percent, several hundreds to several thousand SNP have some correlations to each QTL (assuming that these $\text{r}$ distributions are the same between SNP and QTL).

**Table 1:** Statistics of $\left|\mathbf{r}\right|$ and ${\mathbf{r}}^{2}$ values across the 29 chromosomes in reference populations of 6 French dairy cattle breeds (selection of 1 every 20 SNP within chromosome)

Breeds	Ne (1)	# cows in 2021 reference population	Mean(${\text{r}}^{2}$) (2)	Mean($\left|\text{r}\right|$)	% $\left|\text{r}\right|$ > 0.05	% $\left|\text{r}\right|$ > 0.10	% $\left|\text{r}\right|$ > 0.20
Vosgienne	30	3162	0.0034	0.044	34.2	8.0	0.47
Tarentaise	69	3969	0.0020	0.032	21.0	2.4	0.31
Abondance	56	7671	0.00094	0.020	4.6	0.78	0.28
Normande	93	69,081	0.00066	0.015	1.90	0.66	0.22
Montbeliarde	86	214,985	0.00067	0.015	1.91	0.72	0.25
Holstein	95	377,996	0.00065	0.015	2.71	0.67	0.21

(1) Ne estimate for each breed, based on genealogical data (from [17])

(2) statistics based on 2,876,401 to 3,640,951 r-values per breed

The following section addresses the impact of long-distance associations on the part of GEBV variance explained by the different classes of QTL-SNP contributions. In the first analysis, the model included the causal variants (Table 2). The five partial genomic values were obtained by summing the contributions of all SNP-QTL pairs by distance class. With 200 QTL and 13,642 SNP, the number of contributions were 200, 42,660 ± 530, 112,500 ± 1500, 410,000 ± 3700, and 2,156,200 ± 4500 in d = 0, d < 5 Mb, 5 < d < 20 Mb, d > 20 Mb and “other chromosomes” classes, respectively (note these numbers slightly vary according to the QTL simulations). This analysis showed that when the model to predict GEBV includes the causal variants, these variants succeed to capture the largest part of the genetic variability, leaving little contributions to the close markers and nearly zero contributions of distant QTLs.

**Table 2:** Relative contribution (%) of each of the 5 partial genomic values when the QTL are kept in the model (number of QTL = 200)

Class of partial genomic value	Heritability		
	0.3	0.5	0.99
The SNP is the QTL	96.1 (0.5)	97.8 (0.3)	99.75 (0.07)
Distance(SNP,QTL) < 5 cM	3.1 (0.4)	1.7 (0.3)	0.12 (0.04)
5 cM < Distance(SNP,QTL) < 20 cM	0.3 (0.04)	0.2 (0.02)	0.03 (0.01)
Distance(SNP,QTL) > 20 cM	0.4 (0.06)	0.3 (0.04)	0.08 (0.02)
SNP and QTL on different chromosomes	0.1 (0.01)	0.05 (0.01)	0.02 (0.01)

Each partial genomic value is the sum of QTL contributions to SNP effects, according to the distance between QTL and SNP (the SNP is the QTL, QTL-SNP distance < 5 cM, 5-20 cM, > 20 cM, QTL and SNP are on different chromosomes). Mean (standard deviation) over 30 replicates

In the rest the study, we focussed on a more realistic model where the causal variants are excluded from the analysis. Table 3 presents the relative contribution of the partial genomic values according to the number of QTL and the heritability, in a model without residual polygenic effect. The four partial genomic values were obtained by summing the contributions of all SNP-QTL pairs by distance class.

**Table 3:** Relative contribution of each of the 4 partial genomic values according to the number of QTL and the heritability

	Number of QTL								
	100			200			500		
h^2^	0.3	0.5	0.99	0.3	0.5	0.99	0.3	0.5	0.99
< 5 cM	84.9 (2.4)	83.8 (4.1)	81.7 (5.8)	75.7 (4.3)	74.5 (4.0)	73.6 (8.0)	57.0 (4.8)	55.2 (5.7)	58.1 (7.5)
5–20 cM	7.8 (2.5)	7.7 (3.0)	7.4 (3.7)	11.6 (3.2)	11.0 (2.9)	11.7 (4.0)	17.7 (3.3)	17.9 (3.6)	16.6 (4.4)
> 20 cM	2.7 (0.7)	2.9 (1.4)	4.3 (2.4)	4 (1.3)	4.5 (1.4)	5.2 (2.2)	7.8 (2.3)	8.6 (1.6)	8.8 (3.0)
Other chr^a^	4.6 (1.5)	5.6 (2.1)	6.6 (3.2)	8.6 (2.8)	10.1 (3.1)	9.4 (6.1)	17.5 (5.8)	18.2 (4.7)	16.4 (5.8)

Each partial genomic value is the sum of QTL contributions to SNP effects, according to the distance between QTL and SNP (< 5 cM, 5–20 cM,  > 20 cM, other chromosomes)

Mean (standard deviation) over 30 replicates

^a^SNP and QTL chromosomes are different

The percentage of variance explained by the SNP close to the QTL (d < 5 Mb) was the largest (> 80%) when the number of QTL was small (100) but it decreased when the number of QTL increased (up to less than 60% for 500 QTL). In parallel, the contribution of partial genomic values due to QTL distant from the SNP (d > 20 cM or on another chromosome) increased from 7% for 100 QTL to about 25% for 500 QTL. SNP located on other chromosomes explained more variance than SNP at more than 20 Mb on the same chromosome. The effect of heritability was much smaller but we observed a slight and not significant tendency toward a larger contribution of distant QTL when heritability increased.

Table 4 presents the correlations between partial genomic values. In all scenarios these correlations were positive, illustrating the effect of long-distance SNP-QTL association. These correlations increased with the number of QTL and decreased when heritability increased.

**Table 4:** Average correlations between partial genomic values in models according to the heritability and the number of QTL

	NQTL = 100			NQTL = 200				NQTL = 500		
h2 = 0.3	< 5 cM	5–20 cM	> 20 cM	< 5 cM		5–20 cM	> 20 cM	< 5 cM	5–20 cM	> 20 cM
5–20 cM	0.44			0.50				0.59		
> 20 cM	0.16	0.18		0.20		0.20		0.30	0.37	
Other Chrom	0.16	0.11	0.10	0.20		0.15	0.16	0.25	0.21	0.24
h2 = 0.5	< 5 cM	5–20 cM	> 20 cM	< 5 cM		5–20 cM	> 20 cM	< 5 cM	5–20 cM	> 20 cM
5–20 cM	0.37			0.41				0.56		
> 20 cM	0.14	0.19		0.24		0.23		0.26	0.34	
Other Chrom	0.13	0.06	0.10	0.24		0.19	0.18	0.23	0.18	0.25
h2 = 0.99	< 5 cM	5–20 cM	> 20 cM	< 5 cM		5–20 cM	> 20 cM	< 5 cM	5–20 cM	> 20 cM
5–20 cM	0.16			0.27				0.32		
> 20 cM	0.15	0.10		0.07		0.11		0.16	0.14	
Other Chrom	0.10	0.04	0.10	0.19		0.08	0.14	0.17	0.16	0.18

Means over 30 replicates

Table 5 shows a comparison between the models with and without a residual polygenic effect. The residual polygenic component explained 20% of the genetic variance and the heritability varied from 0.3 to 0.99. Because results are quite similar, only the scenario with 200 QTL is presented. The impact of the polygenic effect was limited and not significant. The same pattern was observed with the correlations between partial genomic values, as shown in Table 6. The correlations between partial components were positive both with and without polygenic effects. They were slightly larger with a polygenic effect when the heritability was 0.3 and 0.5, but this difference was not significant, and this trend was less clear for the heritability equal to 0.99.

**Table 5:** Relative contributions (%) of each of the 4 classes of QTL-SNP pairs defined according to their distance

Class of partial genomic value	Without polygenic effect (*)			With a polygenic effect		
	0.3	0.5	0.99	0.3	0.5	0.99
Distance < 5 cM	75.7 (4.3)	74.5 (4.0)	73.6 (8.0)	74.2 (4.5)	73.4 (5.0)	72.6 (7.5)
5 cM < Distance < 20 cM	11.6 (3.2)	11.0 (2.9)	11.7 (4.0)	11.7 (3.0)	11.5 (2.8)	11.4 (5.1)
Distance > 20 cM	4.0 (1.3)	4.5 (1.4)	5.2 (2.2)	4.5 (1.4)	4.7 (1.5)	4.9 (1.7)
SNP and QTL on different chromosomes	8.6 (2.8)	10.1 (3.1)	9.4 (6.1)	9.5 (3.5)	10.5 (4.3)	11.0 (5.9)

Scenarios are without or with polygenic effect, with 200 QTL, and for 3 heritability values. Mean (standard deviations) of 30 replicates

(*) same as in Table 2

**Table 6:** Average correlations between partial DGV in models without or with a polygenic effect

	Without polygenic effect			With a polygenic effect		
**h**^**2**^ **= 0.3**	** < 5 cM**	**5–20 cM**	**> 20 cM**	** < 5 cM**	**5–20 cM**	** > 20 cM**
5–20 cM	0.50			0.51		
> 20 cM	0.20	0.20		0.27	0.31	
Other Chrom	0.20	0.15	0.16	0.22	0.19	0.21
**h**^**2**^ **= 0.5**	** < 5 cM**	**5–20 cM**	**> 20 cM**	** < 5 cM**	**5–20 cM**	** > 20 cM**
5–20 cM	0.41			0.43		
> 20 cM	0.24	0.23		0.24	0.26	
Other Chrom	0.24	0.19	0.18	0.18	0.11	0.09
**h**^**2**^ **= 0.99**	** < 5 cM**	**5–20 cM**	**> 20 cM**	** < 5 cM**	**5–20 cM**	** > 20 cM**
5–20 cM	0.27			0.33		
> 20 cM	0.07	0.11		0.07	0.09	
Other Chrom	0.19	0.08	0.14	0.15	0.09	0.03

Scenario with 200 QTL and three heritability values. Means over 30 replicates

In another scenario, QTL were selected with a MAF ranging from 0.01 to 0.05. Because results are quite similar, only one situation with 200 QTL and h^2^ = 0.3 is presented in Table 7.

**Table 7:** Relative contribution of each of the 4 partial genomic values according to the number of QTL and the heritability, in the case where QTL minor allele frequency is between 1 and 10%

	Number of QTL									
	100			200		500				
h^2^	0.3	0.5	0.99	0.3	0.5	0.99		0.3	0.5	0.99
< 5 cM	87.0 (2.6)	88.2 (2.7)	87.9 (4.0)	81.1 (3.7)	80.7 (3.4)	79.0 (5.8)		67.6 (4.3)	65.5 (5.3)	62.4 (5.5)
5–20 cM	8.4 (2.1)	6.5 (2.3)	4.2 (3.1)	10.5 (3.0)	8.7 (2.7)	7.7 (3.3)		13.5 (2.0)	12.2 (2.3)	12.6 (4.3)
> 20 cM	1.5 (0.5)	1.8 (0.5)	3.0 (1.3)	2.6 (0.7)	3.4 (1.1)	4.7 (1.8)		5.4 (1.4)	6.6 (1.7)	8.2 (2.3)
Other chr*	3.1 (1.0)	3.5 (1.3)	4.9 (1.8)	5.7 (1.7)	7.2 (3.2)	8.6 (3.2)		13.5 (4.3)	15.6 (4.5)	16.8 (5.6)

Each partial genomic value is the sum of QTL contributions to SNP effects, according to the distance between QTL and SNP (< 5 cM, 5-20 cM, > 20 cM, other chromosomes). Mean (standard deviation) over 30 replicates.* SNP and QTL chromosomes are different

Results of methods 1 and 2 applied to the same 2021 Normande female reference population are presented in Table 8. As before, we focused on the first 5 chromosomes and simulated 200 QTLs. Both methods provided regression coefficients of 0.87 and 0.84, significantly lower than one. Results across the 30 simulations were similar, with a standard deviation around 0.03 over the replicates.

**Table 8:** Estimation of erosion factors by Method 1 and Method 2 (h^2^ = 0.3, 200 QTL, 30 replicates)

	$\widehat{\varrho }$	SD($\widehat{\varrho }$)
Method 1	0.84	0.03
Method 2(*)	0.87	0.04

(*) $$\widehat{\varrho }=2 \widehat{{\varrho }_{2}}-1$$

## Discussion

Erosion of genomic values exists in genomic predictions, as widely demonstrated by frequent inflation in validation studies and by the loss in prediction accuracy observed when candidates are more and more distant from the reference populations [13–16]. Our study provides an explanation to this phenomenon, suggesting that it is likely due to long distance linkage disequilibrium, comprising long distance LD between SNP and QTL located on different chromosomes. Even if the average LD between distant loci is close to zero, there is a proportion of correlation coefficients deviating from zero and SNP can catch some part of distant QTL effects. This proportion may be large in small populations, as shown in the regional French breeds. In large populations, this proportion is low but still sufficient to catch a portion of the genetic variance. Indeed, if only a few percent of the distant SNP have some LD with a given QTL, the QTL effect is distributed between a few dozens of close SNP with high (but still incomplete) LD and between several hundreds or thousands of distant SNP with low or moderate LD with the QTL. In the simulated scenarios with 100 to 500 QTL over 5 chromosomes based on a real cattle breed, a portion ranging from 6 to 25% of the variance of the estimated genomic breeding values was explained by contributions of QTL distant from the SNP. Results were unchanged when QTL were sampled with a rather low MAF, which corresponds to a likely more realistic situation.

One may argue that long distance LD should disappear over time. A basal LD is a function of the effective population size Ne (of the whole population or, more importantly, of the reference population for the genomic evaluation) and remains more or less constant across generations on the average LD at the genome level. However, this is not true for a given pair of loci. Two opposite forces explain this steady-state LD: (1) The existing long-range LD is reduced by a factor 0.5 in each generation due to segregations; and (2) LD is regenerated by drift (and possibly selection) at random. Therefore, the LD between 2 unlinked loci existing in the reference population reduces by a factor 0.5. Simultaneously, new LD is created at random in the next generation for other loci, in such a way, while overall changes are negligible, that individual values between pairs of loci may vary considerably.

Another possible reason for this pattern lies in the strong shrinkage of the effects of markers situated close to the QTL due to the influence of the prior information. Indeed, with GBLUP or SNP-BLUP (or their extension to Single Step) model, all markers receive the same prior variance, and the parameter $(\lambda =\frac{{\upsigma }_{\text{e}}^{2}}{{\upsigma }_{\text{s}}^{2}})$ has a relatively high value, thus dominating the diagonal elements of the matrix $\mathbf{M}\mathbf{^{\prime}}\mathbf{M}$, except for sufficiently large reference populations. Consequently, the estimated effects of markers close to the QTL may experience substantial shrinkage and are smaller than the true QTL effect, even if considered altogether. As a result, the unexplained part of the true QTL effect becomes available to be captured by distant markers, potentially leading to their contribution to the estimated genomic value. Under this assumption, we could expect a stronger effect of long-distance LD when the number of QTL is small (i.e., when ${\upsigma }_{\text{q}}^{2}\gg {\upsigma }_{\text{s}}^{2}$). However, with the already large Normande reference population used in this study (~ 69,000 individuals), the strongest long-distance LD impact was observed with a large number of QTL. Therefore this expected effect of the prior information was not observed and we cannot confirm our initial assumption. Another factor can explain this discrepancy: the relationship between the reference and the candidates decreases when the number of QTL increases, as shown by [21], and this property is likely to be more important than shrinkage consequences. Nonetheless, it is important to note that when the size of the reference population is very large, the influence of prior information decreases, and this assumed pattern is likely to gradually decrease and disappear.

The influence of the covariance structure of SNP effects is illustrated in practice in Table 9, corresponding to the French Holstein 2021 Single Step evaluation for resistance to paratuberculosis [22]. This trait has a low heritability (0.15) and a rather limited reference population compared to most other traits measured in the same breed (56,766 cows with phenotypes (after filtering), but only 12,431 cows or sires with genotypes). Table 9 illustrates that the sum of individual SNP effect variances explains a very small proportion of the genomic variance (1%), whereas the sum of haplotype effect variances gradually increases with haplotype size, showing the huge amount of positive covariances between marker effects. Even when entire chromosomes are considered, the sum of the 29 variances represents only 58% of the total, showing that 42% of the total variance originate from inter-chromosomal covariances. These positive covariances are due to long distance LD. This very high value may be surprising but this quite extreme situation likely results from the limited size of the reference population in only one French region and the rather low heritability of the trait.

**Table 9:** Proportion of genomic estimated breeding values (GEBV) variance explained by haplotypes of various size in the resistance to paratuberculosis evaluation of the French Holstein breed (from [22])

Interval length (# markers)	1 (single markers)	10	100	500	Entire Chromosomes	Whole genome
% variance explained	1	8.5	26	47	58	100
# intervals	53,469	5360	548	121	29	1

Variances were computed from partial GEBV per interval in the evaluated population

Our results show that the inclusion of a polygenic effect in the model does not result in a smaller proportion of variance explained by distant markers and by markers located on different chromosomes. At first sight this result may seem counterintuitive as one could expect, and it is frequently argued, that the polygenic effect would account for these long-distance effects since it captures the genetic relationships between individuals [11]. In our simulation, distant SNP as well as SNP in other chromosomes altogether explained a similar or slightly higher proportion of the genomic variance in the model with a polygenic effect. In parallel, the variance share due to close markers (d < 5 Mb) slightly decreased, and the correlations between SNP effects were a bit higher in the model including a polygenic effect, suggesting an increased influence of distant markers. However none of these differences were significant. As previously, a possible interpretation of the lack of improvement with the polygenic component is the shrinkage of estimated SNP effects. Indeed, in the presence of a polygenic effect with variance ${\upsigma }_{\text{u}}^{2}$, the total variance due to SNPs is reduced to ${\upsigma }_{\text{g}}^{2}$ − ${\upsigma }_{\text{u}}^{2}$ and results in an increased variance ratio $(\lambda =\frac{{\upsigma }_{\text{e}}^{2}}{{\upsigma }_{\text{s}}^{2}})$. It can thus be concluded that the inclusion of a polygenic effect in the model should be motivated primarily by the need to account for the genetic variance not captured by the SNPs, rather than as a means to reduce inflation. Indeed, in a model with a polygenic effect, the breeding value is the sum of SNP effects and of the residual polygenic effect. For a candidate with no phenotypic information, the estimated Mendelian sampling term has an inflated SNP component and a zero (i.e., strongly deflated) polygenic component, possibly resulting in a non-inflated prediction. However this arbitrary combination affects all individuals, whether in the reference population or not, and all young candidates are equally affected irrespective to their distance to the reference. Therefore this model may reduce the accuracy of the genomic prediction if the assumed polygenic variance is too high.

A deterministic prediction of the erosion factor over one generation is highly desirable. Cuyabano et al. [19] proposed a method providing an upper bound of the accuracy of the prediction of the candidates, a parameter directly related to erosion. Erosion likely depends on the Ne, the relationship within the reference population, the heritability of the trait and its genetic architecture (i.e., the number of QTL). Even if candidates have parents in the reference population, the distance to the “barycentre” of the reference population, i.e. the relationship between the candidate and the reference population, may vary (e.g., when the reference population includes one or several generations, one or several strains or breeds, etc.), and this average relationship is also a factor of variation. The erosion factor depends on both the population and the trait, since different traits present different heritabilities and the reference population (even in the same breed) will usually vary according to the trait, based on the phenotyped individuals.

In the present study, we proposed two simulation-based approaches to address the erosion phenomenon and to estimate $\varrho$, while both of these approaches are practical and effective. Results likely depend on the genetic architecture assumed for the QTL. In the example presented in Table 6, we assumed 200 QTL over 5 chromosomes, for a total heritability of 0.3. These choices were quite arbitrary and future studies should further explore different values for these parameters. Scaling up to the whole genome while maintaining the same heritability means a smaller QTL variance, more across chromosomes comparisons and probably a stronger erosion factor. This topic also requires further investigation.

Method 1 is quite straightforward to implement, as it involves simulating one additional generation and estimating expected SNP effects with both the reference population and the new generation using standard software. In contrast, Method 2 requires a specific program to compute all the QTL contributions to SNP effects and then to erode them according to the genetic map. Nevertheless, Method 2 provides an explicit biological basis to understand and interpret erosion across generations. Both methods assume that genotypes are phased without errors to properly simulate recombination events.

Method 1 generates progeny from pairs of parents, leading to erosion on both sire-progeny and dam-progeny pathways. Method 2, on the other hand, simulates erosion through recombination at only one meiosis, resulting in the erosion factor estimated by method 2 being half of that estimated by method 1. This scale difference must be considered when interpreting results.

Additionally, method 2 considers the whole reference population whereas method 1 generates a new generation based on assumptions about the number and choice of parents sampled. Therefore, results between the two methods are not equivalent and likely exhibit differences.

In the example provided with the Normande breed and 200 QTL, the erosion factor was around 0.85 over one generation, which is close to the inflation factor found in different studies, e.g. [13–16]. This means that the best selection candidates are overestimated by approximately 15% when they receive their first genomic evaluation. Analogously we can assume that the worst selection candidates are underestimated by the same amount. Therefore this information is important to take into account in practical selection.

Erosion has important practical consequences in selection and it is critical to minimize its impact. Several pieces of solution can be envisioned. A first possibility is to keep the reference population close to the candidates to reduce erosion [5]. Therefore, removing the oldest generations is probably valuable and the best trade-off must be found between erosion and quantity of information [23]. We also have to remember that keeping the most recent generation(s) in the reference increases relationship (measured by $\mathbf{M}\mathbf{M}\mathbf{^{\prime}}$) and therefore long distance LD (measured by $\mathbf{M}\mathbf{M}$) within the reference, therefore there may be an optimum number of generations in the reference. Whereas sires and maternal grandsires are generally genotyped, it is not always the case for dams. We encourage to genotype both parents to reduce erosion. Regarding acceleration of selection, breeders frequently tend to decrease generation interval to benefit from cumulated selection intensity assuming no loss in accuracy. If the parents do not have phenotypes, the distance between the candidates and the reference population is increased, thus increasing inflation of the estimated genomic values. In practice, the best candidates are therefore overestimated and show disappointing results when confirmation phenotypes become available. This risk must be accounted for when applying accelerated selection. The best option is to predict the individual genomic values accounting for erosion to optimise this accelerated process.

All our results were obtained with GBLUP, which assumes constant SNP variance. An increased marker density over the whole genome combined with GBLUP has been shown to increase accuracy only marginally (e.g., [9]) and probably does not reduce the long-distance LD effect. Alternative models exist using the full Bayesian alphabet [1, 8, 24, 25] and allow the appropriate variance to be assigned to each SNP. With a higher marker density than in the present study, these models are able to select the causal variants or SNPs very close to the causal variants and eliminate the other SNPs. If successful, these models should eliminate at least some of the long-range LD effects. In general, models that include causal variants or SNPs close to causal variants have been shown to be less inflated than GBLUP [16, 26–28]. Despite their advantages, these methods are however still rarely used in routine evaluation, mainly because they require individual parameterisation and fine tuning for each population and each trait [29]. However, GWAS combined with genome annotation will unravel more and more candidate causal variants, and we expect future genomic evaluation systems to increasingly incorporate this biological information. This would ensure a lower dependency on the distance to the reference population.

As present genomic evaluations still rely on GBLUP, erosion must be accounted for to keep unbiased predictions. In the absence of a method to directly include the erosion into the mixed model equations, its implementation can be done by post-processing the genomic breeding values. Here, we consider that, by definition, individuals in the reference population are assumed to possess non-eroded SNP effects. It is worth noting that this assumption may warrant discussion due to potential heterogeneity within the reference population. The estimated SNP effect represents an expectation and may not align with individual situations. However, this point goes beyond the scope of this study. Erosion primarily concerns selection candidates, i.e., genotyped individuals without phenotype data and, therefore, animals that are not included in the reference population. When their parents, referred to as s and d, are part of the reference population, we assume that the parent’s average (PA) genomic value, denoted as

$$\text{PA}=0.5 \left(\widehat{{\text{g}}_{\text{s}}}+ \widehat{{\text{g}}_{\text{d}}}\right),$$

remains unaffected. Indeed, their estimated genomic values are based on performances; and this is especially the case for sires with progeny evaluation, and therefore with very reliable proofs. Erosion influences the deviation from PA, *i.e.*, the predicted Mendelian sampling term. We propose applying the following formula:

8 $${\widehat{\text{g}}}_{\text{i }(\text{eroded})}=\text{PA}+{ \varrho }\left({\widehat{\text{g}}}_{\text{i}}-\text{PA}\right),$$

with ${\varrho }$ being the erosion factor.

When the parents of a candidate are not in the reference population, erosion applies for each generation between the reference population and the candidate. Following a similar approach as described by [18], the number of generations between the candidate and its closest relatives within the reference population is determined on both the sire and dam pathways and k is their sum. Erosion can then be considered in estimated genomic values using Eq. (9):

9 $${\widehat{\text{g}}}_{\text{i }(\text{eroded})}=\text{PA}+ {\varrho }^{\text{k}/2} \left({\widehat{\text{g}}}_{\text{i}}-\text{PA}\right).$$

The computation of k raises the question of the distance (in number of generations) between the parents (assumed to belong to the reference population) and the barycentre of this reference population. A possible solution would be to add to k a term corresponding to this distance, but how to compute this term requires further investigation.

When the parents themselves are candidates, *i.e.,* genotyped and not in the reference population, erosion also applies to them. This formula is applied recursively, processing parents before progeny, such that erosion of the parents affects the PA of their progeny. This recursive approach highlights that the DGV of candidates born from very young parents experience significant erosion, aligning well with practical observations [5, 12]. Therefore, breeding schemes with accelerated generations without updating reference data tend to accumulate more erosion and may be less appealing than initially anticipated. As mentioned before, erosion tends to reduce effective cumulated selection differential and must be accounted for when optimising this accelerated process.

As shown by [18], erosion not only affects breeding value prediction but also their reliability, however with a coefficient equal to ${\varrho }^{2}$ instead of $\varrho$. Dekkers et al. [18] considered physical recombination whereas we extend the concept to any kind of linkage disequilibrium including long distance. While erosion factors were typically over 0.97 in their study, we find a much lower value. The loss in genomic accuracy is therefore very fast. Theoretical accuracies calculated based on the inverse of the coefficient matrix ignore erosion and tend to overestimate the reliabilities for candidates. Therefore, these reliabilities must be adjusted accordingly. Let us illustrate this situation with a hypothetical example where the genomic reliability in the reference population is ${\text{R}}_{\text{g}}^{2}$. Candidate animal i is born from sire s and dam d, with polygenic reliability ${\text{R}}_{\text{ps}}^{2}$ and ${\text{R}}_{\text{pd}}^{2}$, resulting in pedigree-based reliability of ${\text{R}}_{\text{pi}}^{2}=0.25 ( {\text{R}}_{\text{ps}}^{2} + {\text{R}}_{\text{pm}}^{2})$. The genomic reliability of candidate i is ${\text{R}}_{\text{gi}}^{2}= {\varrho }^{k} {\text{R}}_{\text{g}}^{2}$. Ignoring some (limited) double counting, the total reliability of the candidate can be estimated by ${\text{R}}_{\text{i}}^{2}= \frac{{\text{R}}_{\text{pi}}^{2}+ {\text{R}}_{\text{gi}}^{2}-2 {\text{R}}_{\text{pi}}^{2} {\text{R}}_{\text{gi}}^{2}}{1- {\text{R}}_{\text{pi}}^{2} {\text{R}}_{\text{gi}}^{2}}$ [30, 31]. A numerical example of the loss in reliability due to erosion is presented in Table 10.

**Table 10:** Illustration of the loss in candidate’s reliability due to erosion in three pedigree situations (genomic reliability ${\mathbf{R}}_{\mathbf{g}}^{2}=0.6$ in the reference, erosion factor $\boldsymbol{\varrho }=0.9$)

Status of the candidate	Candidate’s polygenic pedigree reliability	Genomic reliability in the reference	Candidate’s reliability without erosion	Distance of the candidate to the reference (k)	Eroded genomic reliability of the candidate	Candidate’s reliability after erosion	Loss in reliability due to erosion
(1)	0.35	0.6	0.67	2	0.49	0.60	0.07
(2)	0.35	0.6	0.67	3	0.44	0.57	0.10
(3)	0.35	0.6	0.67	4	0.39	0.54	0.13
(4)	0.15	0.6	0.63	4	0.39	0.45	0.17

(1) Sire and dam in the reference, with genotypes and phenotypic information. (2)Sire and maternal grandsire in the reference, dam not genotyped but with phenotype. (3) Sire and dam not genotyped but with phenotypic information. (4) Sire and dam without phenotypic information (accelerated selection)

## Conclusion

In this study we showed that erosion exists in genomic evaluation, even when both parents belong to the reference population. We provided a biological interpretation related to long distance LD, and proposed practical approaches to measure the erosion factor. When considering the overall prediction of selection candidates, contributions from short-distance LD tend to remain relatively stable because they are only mildly eroded by recombination [18]. However, contributions from long-distance LD are halved each generation. The extent of erosion varies with the relative weight of short and long-distance LD, but it should never be disregarded.

Further investigations are needed to theoretically determine the erosion factor $\varrho$. Nonetheless it is clear that this factor increases with baseline LD in the population, and therefore decreases when effective population size (Ne) and genome length (L) increase. The structure of the reference population is also important, especially when individuals are more related than in the overall population. Additionally, erosion is likely influenced by the genetic architecture of the traits (such as the number of QTL and magnitude of their QTL effects) and the model used (SNP-BLUP/GBLUP vs Bayesian models, the latter being less affected by erosion).

It is also relevant to explore the impact of the structure of the reference population, such as its heterogeneity in terms of time span, selection, and relationship. While the proposed methods to assess erosion consider the smallest distance from the candidate to the reference population, alternative approaches using the barycentre of the reference population warrant investigation.

However, one can anticipate that:

1. Inflation factors, frequently observed between 0.8 and 0.9, give the magnitude of the erosion phenomenon;
2. Models including causal variants tend to be more persistent and less subject to erosion, as demonstrated;
3. Even if models that incorporate a residual polygenic component may appear to have less inflated predictions for candidates, the polygenic effect does not capture long-distance LD effects and, therefore, does not improve predictions in terms of persistence. We believe that accounting for erosion is a more rigorous and accurate approach, even if it requires post-processing.

## Data Availability

Raw genotypes are part of a reference population used for genomic selection and have commercial value. Therefore, restrictions apply to their availability and they are not publicly available. The authors can be contacted for a reasonable request.
